# Perceptions and experiences of undergraduate medical students regarding social accountability: a cross-sectional study at a Subsaharan African medical school

**DOI:** 10.1186/s12909-024-05412-3

**Published:** 2024-04-12

**Authors:** Lorraine Oriokot, Ian Guyton Munabi, Sarah Kiguli, Aloysius Gonzaga Mubuuke

**Affiliations:** 1 Department of Pediatrics and Child Health, Makerere University College of Health Sciences, Kampala, Uganda; 2 Department of Anatomy, Makerere University College of Health Sciences, Kampala, Uganda; 3 Department of Radiology, Makerere University College of Health Sciences, Kampala, Uganda

**Keywords:** Social accountability, Medical school, Medical education, Medical students, Community-based education research and service

## Abstract

**Background:**

Medical schools are called to be socially accountable by medical education and healthcare system stakeholders. Social accountability is a feature of excellent medical education. Medical students are essential to the development of socially accountable medical schools. Therefore, understanding the perceptions and experiences of medical students regarding social accountability is critical for efforts to improve social accountability practices and outcomes.

**Methods:**

This cross-sectional online questionnaire-based survey used Google Forms and involved medical students in their fourth and fifth years of study at the Makerere University School of Medicine. The survey was conducted between September 2022 and October 2023. We used a study questionnaire and a validated toolkit designed by students as part of The Training for Health Equity Collaborative to gauge a school’s progress towards social accountability in medical schools to collect data on demographics, perceptions and experiences and evaluate social accountability.

**Results:**

Out of 555 eligible medical students, 426 responded to the online questionnaire. The response rate was 77%. The mean age of the students was 25.24 ± 4.4 years. Almost three fourths of the students were male (71.3%), and slightly less than two thirds were in their fourth year of study (65%). Almost half of the students (48.1%%) evaluated the school as doing well with regard to social accountability. The evaluation items referring to community-based research and positive impact on the community had the highest mean scores. Only 6 (3.6%) students who reported hearing of social accountability had a clear understanding of social accountability. Students receiving career guidance in secondary school was associated with evaluating social accountability in the medical school as strong (p-0.003).

**Conclusions:**

Medical students evaluated the medical school favorably forsocial accountability despite lacking a clear understanding of social accountability. Receiving career guidance in secondary school was significantly associated with a positive evaluation of social accountability.

## Background

Medical students are essential to the development of a socially accountable medical school [1, 2]. Social accountability for medical schools is defined as the obligation of the medical school to direct education, research, and service activities toward the most important needs of the community served by the medical school and its graduates [3]. While medical students are expected to learn about social accountability and become socially accountable practitioners, little has been documented about their perceptions and experiences of social accountability.

Medical students have distinct and unique experiences during training, which may influence their future practice and choices following graduation [4, 5]. It is important to understand these experiences to improve the teaching and learning of social accountability. Previous studies have shown that medical students have a limited understanding of social accountability [5–7]. The partnership pentagram identifies five key stakeholders for social accountability in health professions education including policymakers, health administrators, health professionals, academics and community members [2, 8]. A survey of Deans in Korea reported that interactions between partners had the greatest influence on social accountability in medical education [9]. Medical students are the future of the healthcare system; therefore, understanding their perceptions and experiences could be a starting point for improving their learning and adoption of social accountability [1, 4].

The Students’ Toolkit on Social Accountability of Medical Schools was developed through a collaboration between the International Federation of Medical Students Associations (IFMSA) and the Training for Health Equity Network (THEnet) [1, 4, 10]. The toolkit aims to provide a brief introduction to social accountability for medical students and empower them to make a difference in their schools in the area of social accountability. The toolkit is an evaluation tool for students to assess the progress made by their medical school in terms of social accountability and to create action plans to improve social accountability at the medical school [1]. This kit has been applied in various settings most often in high-income countries [4, 11].

The perceptions and experiences of medical students regarding social accountability are unique because of contextual differences that may influence their learning and adoption of social accountability [12, 13]. There is a dearth of published literature about these perceptions and experiences, particularly from sub-Saharan Africa. The purpose of this study is to determine the perceptions and experiences of medical students regarding social accountability at the Makerere University School of Medicine.

## Methods

### Study design and questionnaire

We conducted a cross-sectional online questionnaire-based survey between September 2022 and October 2023 using Google Forms. This study collected responses from medical students from the Makerere University School of Medicine. The validated Students’ Toolkit on Social Accountability in Medical Schools, which has been applied widely to study perceptions of social accountability, was used for this study, with some modifications [1]. The final study questionnaire had three sections: demographic information, perceptions, and experiences related to social accountability; and the evaluation section from the toolkit. The questionnaire was pretested on eight undergraduate medical students in their fourth or fifth year of study. The link to the questionnaire on Google Forms was sent to each student’s email. Each link was unique to each email address to avoid reuse and double enrollment.

### Study setting

We conducted this study at the Makerere University School of Medicine in Uganda. Makerere University is a government-owned university, and the Makerere University School of Medicine is the oldest medical school in East Africa. The Bachelor of Medicine and Bachelor of Surgery (MBCHB) program spans five years. In their third and fourth year of study, medical students spend time in the community for Community Based Education, Research and Service (COBERS). Before COBERS, students undergo preparatory sessions at the university and are assigned site and university supervisors to follow their progress. During COBERS, students identify a community problem, develop and implement interventions. Thereafter, the students evaluate the impact of the intervention [14].

### Characteristics of participants

We invited all students in their fourth or fifth year of medical school to participate in the study through trained research assistants. A total of 555 students were eligible to participate in the study. We selected fourth- and fifth-year students because they were more likely to have had experiences related to social accountability in medical school. We identified twelve research assistants from among the fourth- and fifth-year students and trained them in the study procedures. The research assistants obtained written informed consent from the participants and registered their email addresses. We sent links to the electronic survey to the registered email addresses. The research assistants provided weekly phone call or in-person reminders to students who had not responded to the survey. We collected responses over two academic years therefore two sets of fourth year students were enrolled. However, the fifth years in the second academic year of the study had already been enrolled as fourth year students.

### Statistical analysis

We collected the data using Google Forms. We exported the data to Microsoft Excel, checked for completeness and missing data. We then cleaned the data. The data were analyzed with R statistical software. The descriptive statistics are reported. The total score for the students’ toolkit 12 social accountability evaluation items was computed, and the means and standard deviations for the individual items are presented. The total scores were categorized according to the key provided in the student toolkit. The categories included 0–8, weak foundation; 9–17, some evidence of social accountability; 18–26, doing well (identify areas of improvement); and 27–36, strong foundation. For the regression analysis to determine the factors associated, the categories defined in the toolkit were classified in two groups (1) Limited social accountability (weak and some social accountability) and (2) Strong social accountability (looking for areas of improvement and strong social accountability). The chi-square test and Fisher’s exact test were used. A *p* value < 0.05 indicated statistical significance.

## Results

There were 555 eligible medical students at the Makerere University School of Medicine during the study period. This study involved 426 medical students therefore the response rate was 77%. The mean age of the students was 25.24 ± 4.4 years. Most of the participants were in their fourth year of study, as the link was available over two academic years. The characteristics of the study participants are summarized in Table 1.

**Table 1:** Characteristics of the study participants

Variable	Characteristic	*N* = 426 (%)	(95 CI)1–2
Gender	Female	122 (28.64)	(24.44–33.23)
	Male	304 (71.36)	(66.77–75.56)
Age	20–24	283 (66.43)	(61.70–70.86)
	25–30	86 (20.19)	(16.54–24.38)
	31–35	32 (7.51)	5.272–10.55)
	36–47	25 (5.87)	(3.341–7.833)
Tribe	Muganda	158 (37.26)	3.910–8.656)
	Munyankole	51 (12.03)	(9.164–15.60)
	Other tribe	215 (50.71)	(45.85–55.56)
Nationality	Ugandan	410 (96.24)	(93.84–97.77)
	Other Nationality	16 (3.76)	(2.235–6.156)
Highest education level before medical school	A level and equivalent	317 (74.41)	(69.94–78.44)
	Bachelor	36 (8.45)	(6.066–11.61)
	Diploma	73 (17.14)	(13.75–21.13)
Year of study	Year 4	276 (64.79)	(60.02–69.29)
	Year 5	150 (35.21)	(30.71–39.98)
Received career guidance in secondary school	Yes	343 (80.71)	(76.56–84.28)
	No	82 (19.29)	(15.72, 23.44)

### Student perceptions and experiences of social accountability

**Fig. 1:**
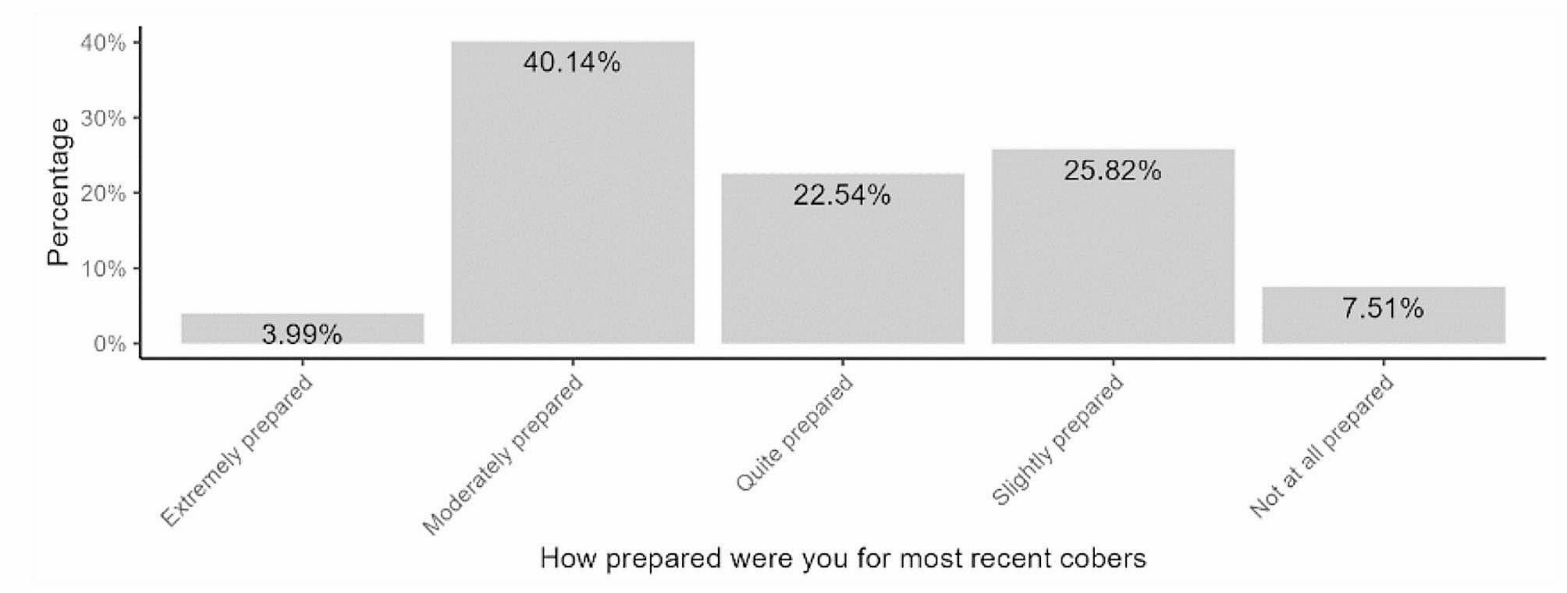
Level of preparedness for last community-based education research and service experience

When asked if they had ever heard about social accountability, 165 (38.73%) students responded ‘yes’. To build on this previous question, participants were asked ‘What do you understand by the term social accountability?’. Only 6 (3.6%) of those who reported having heard about social accountability clearly defined the term capturing the active and multidimensional nature of social accountability. Of the 165 (39%) participants who reported hearing about social accountability before, 91 (55%) encountered the term in personal reading. The average time spent in community-based education research and service was 6.8 weeks, and most (40.1%) of the students reported feeling moderately prepared for their last COBERS experience. (Fig. 1)

### Students’ evaluation of social accountability at medical schools

**Fig. 2:**
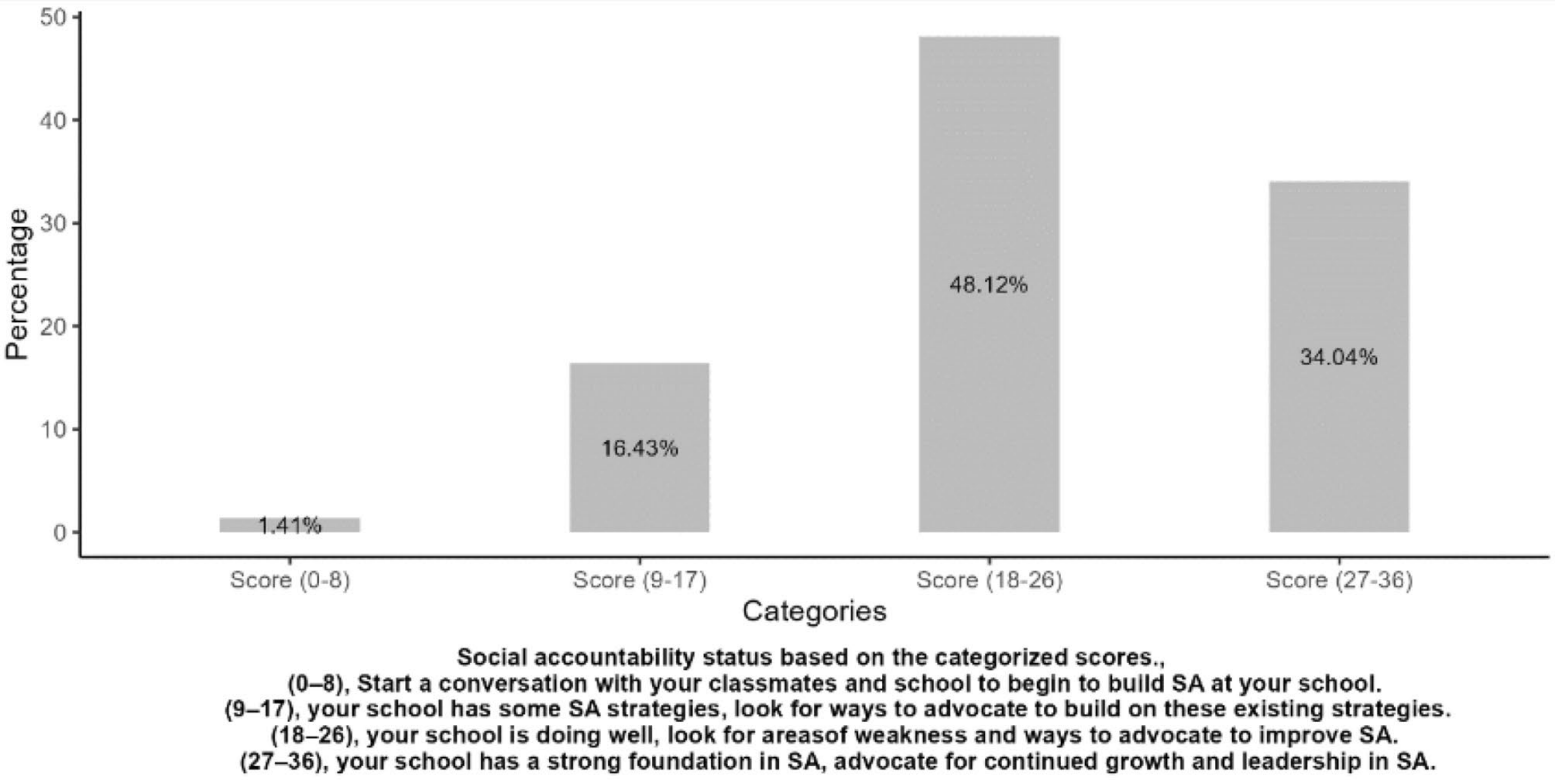
Evaluation categories of social accountability at the medical school

Using the Students’ Toolkit for Social Accountability in Medical Schools, 48.1% of the medical students evaluated the medical school as doing well in social accountability, with a total score between 18 and 26. In contrast, 1.4% of the students felt that the medical school had a weak foundation for social accountability. (Fig. 2)

Of the twelve items used to assess social accountability, seven items (1, 2, 3, 7, 8, 11 and 12) were related to perceptions, while five items (4, 5, 6, 9 and 10) were related to experiences. Item 10 (Does your school have community-based research? ) 2.57 ± 0.62 and Item 12 (Does your school have a positive impact on the community? ) 2.434 ± 0.67 had the highest mean scores. Item 4 (Do you learn about other cultures? ) 1.37 ± 0.93 and Item 8 (Do your teachers reflect the sociodemographic characteristics of the reference population? ) 1.60 ± 0.92 had the lowest mean scores. (Table 2)

**Table 2:** Students’ evaluation of social accountability at the Makerere university school of medicine

Question	Excellent	Good	Somewhat	No	Mean ± SD
Does your institution have a clear social mission statement around the communities that they serve?	91 (21.4)	186 (43.7)	109 (25.6)	40 (9.4)	1.77 ± 0.89
Does your curriculum reflect the needs of the population you serve?	99 (23.2)	216 (50.7)	89 (20.9)	22 (5.2)	1.92 ± 0.80
Does your school have community partners or stakeholders who shape your school?	87 (20.4)	164 (38.5)	120 (28.2)	55 (12.9)	1.66 ± 0.94
Do you learn about other cultures and other social circumstances in medical context in your curriculum?	53 (12.4)	133 (31.2)	157 (36.9)	83 (19.5)	1.37 ± 0.93
Do the places/locations you learn at in practice include the presence of the populations that you will serve?	161 (37.8)	174 (40.9)	74 (17.4)	17 (4.0)	2.12 ± 0.84
Are you required to do community-based learning (opposed to only elective opportunities)?	211 (49.5)	146 (34.3)	44 (10.3)	25 (5.9)	2.28 ± 0.87
Does your class reflect the sociodemographic characteristics of your reference population?	101 (23.7)	175 (41.1)	99 (23.2)	51 (12.0)	1.77 ± 0.95
Do your teachers reflect the sociodemographic characteristics of your reference population?	71 (16.7)	170 (39.9)	129 (30.3)	56 (13.2)	1.60 ± 0.92
Does your learning experience also provide an active service to your community?	145 (34.0)	193 (45.3)	76 (17.8)	12 (2.8)	2.11 ± 0.79
Does your school have community-based research?	270 (63.4)	132 (31.0)	21 (4.9)	3 (0.7)	2.6 ± 0.62
Does your school encourage you to undertake generalist specialties (e.g. family medicine, general practice)?	153 (35.9)	123 (28.9)	94 (22.1)	56 (13.2)	1.88 ± 1.05
Does your school have a positive impact on the community?	225 (52.8)	164 (38.5)	34 (8.0)	3 (0.7)	2.43 ± 0.67

The only factor that was significantly associated with evaluation of social accountability in medical school as strong was receiving career guidance in secondary school (p 0.003). (Table 3)

**Table 3:** Factors associated with students’ evaluation of social accountability

Variable	Characteristic	Overall, *N* = 426	Limited SA, *N* = 76	Strong SA, *N* = 350	*p* value
Gender	Female	122	28 (23.0)	94 (77.1)	0.081
	Male	304	48 (15.8)	256 (84.2)	
Receiving career guidance in secondary school	Yes	344	52 (15.1)	292 (84.9)	0.003
	No	82	24 (29.3)	58 (70.7)	
Year of study	Year 4	276	47 (17.0)	229 (83.0)	0.6
	Year 5	150	29 (19.3)	121 (80.7)	
Age category	19–24	283	52 (18.4)	231 (81.6)	0.7
	25–47	143	24 (16.8)	119 (83.2)	
Nationality	Ugandan	410	75 (18.3)	335 (81.7)	0.3
	Other	16	1 (6.3)	15 (93.8)	
Education level prior to medical school	A-level and equivalent	317	57 (18.0)	260 (82.0)	0.9
	Graduate	109	19 (17.4)	90 (82.6)	
CGPA category	2.36–3.99	352	61 (17.3)	291 (82.7)	0.5
	4.00–5.00	67	14 (20.9)	53 (79.1)	

## Discussion

We evaluated the perceptions and experiences of medical students at the Makerere University School of Medicine regarding social accountability. Medical students are central to the adoption and practice of social accountability efforts in medical schools. The perceptions and experiences of the medical students in this study reflect exposure to the concept and practice of social accountability. Learning social accountability requires deliberate and meaningful efforts in which medical students are considered partners. Our study findings suggest that medical students at the Makerere University School of Medicine have varied perceptions and experiences of social accountability; most of the students evaluated the medical school favorably, and receiving career guidance in secondary school was associated with a positive evaluation of social accountability. Most medical students felt that the medical school was doing well in social accountability and needed to look at areas of weakness and ways to advocate for improving social accountability. The recommended actions for students to take concerning each scoring category of social accountability in the student toolkit reflect the continuous quality improvement approach that is vital for social accountability [1]. The highest mean scores for items in the students’ toolkit for social accountability in medical schools were for community-based research and for the positive impact of the medical school on the community. The least favorable assessment items include learning about other cultures and teachers reflecting the reference population. Medical students come from varied backgrounds and have different experiences during medical education; therefore, it is not surprising that the perceptions, experiences and evaluations of the medical school were diverse. While career guidance in secondary school was associated with a good evaluation of social accountability at the medical school, we cannot confirm a causal relationship between the two. This relationship may be an area for further evaluation. This study helps us to characterise medical students’ views regarding social accountability and the factors that may influence these views. These findings provide a starting point for improving student experiences of social accountability in medical education.

The high percentage of students who gave a good evaluation of social accountability at medical school reflects efforts by the medical school to achieve this goal. These efforts include community-based education, research and services and adopting a competency-based medical education curriculum to better meet the community’s needs [15, 16]. Our findings are comparable to those of a study conducted at a Saudi Arabian government-funded medical school where most students felt that the medical school was performing well in terms of social accountability [4].

There have been gains in pursuing social accountability goals, and more needs to be done to enable students to understand the concept and demonstrate its values. Our findings concur with a previous study that showed a poor understanding of social accountability among stakeholders. This previous study also provided evidence of social accountability in medical school activities [6]. Similarly, a qualitative study in the United Kingdom also showed that students did not understand the concept of social accountability or feel that it has implications for their medical education or future practice [5].

The major strength of this study lies in the relatively large sample size, as we tried to enroll all eligible participants. The limitation of this study is that it was conducted at one study site, which may limit the generalizability of the findings. However, the presence of similar findings in other settings supports the generalizability of our findings. Recall bias was minimized by enrolling students who were still in medical school.

## Conclusions

Medical students at the Makerere University School of Medicine have varied perceptions and experiences of social accountability. However, the students lack a clear understanding of social accountability. Most students felt moderately prepared for COBERS and evaluated the medical school positively for social accountability. Regarding individual items in the student toolkit, the presence of community-based research and the school having a positive impact on the community had higher mean evaluation scores. Receiving career guidance in secondary school was associated with a positive evaluation of the medical school.

## Recommendations

The medical school should provide students with more opportunities to learn about social accountability and routinely evaluate the perceptions and experiences of medical students regarding social accountability. In addition, the medical school should effectively prepare students for Community-Based Education Research and Service.

## Data Availability

The datasets used and/or analyzed during the current study are available from the corresponding author upon reasonable request.

## Abbreviations

COBERS: Community Based Education Research and Service
